# Endoplasmic Reticulum Stress PERK-ATF4-CHOP Pathway Is Associated with Hypothalamic Neuronal Injury in Different Durations of Stress in Rats

**DOI:** 10.3389/fnins.2017.00152

**Published:** 2017-03-24

**Authors:** Shanyong Yi, Weibo Shi, He Wang, Chunling Ma, Xiaojing Zhang, Songjun Wang, Bin Cong, Yingmin Li

**Affiliations:** Laboratory of Forensic Medicine, Department of Forensic Medicine, Hebei Key Collaborative Innovation Center of Forensic Medical Molecular Identification, Hebei Medical UniversityShijiazhuang, China

**Keywords:** hypothalamus, HPA axis, glucocorticoid, stress, PERK-ATF4-CHOP pathway

## Abstract

The hypothalamus, which is the initial part of the hypothalamic-pituitary-adrenal (HPA) axis, plays a critical role in regulating stress in the central nervous system. The present study aimed to determine whether endoplasmic reticulum stress in hypothalamic neurons is differentially stimulated by varying durations of stress exposure, which ultimately leads to pathological changes in neurons by affecting HPA axis function. There is a need for better morphological evidence of the mechanisms involved in stress-induced neuron injury. A stress model was established in rats by restraining for 8 h and forced ice-water swimming for 5 min each day. The stress-inducing process lasted for 1, 3, 7, 14, and 21 days. Enzyme-linked immunosorbent assay (ELISA) was used to assay serum glucocorticoid levels. Thionine staining was used to observe morphological changes in hypothalamic neurons. Immunohistochemistry and microscopy-based multicolor tissue cytometry (MMTC) was used to detect changes in expression of endoplasmic reticulum stress protein GRP78, ATF4, and CHOP. Serum glucocorticoid levels significantly increased after 3 days of stress exposure and the levels peaked by 7 days. By 21 days, however, the levels were significantly decreased. Thionine staining revealed that prolonged stress exposure resulted in hypothalamic neurons with edema, a lack of Nissl bodies, and pyknotic neurons. Immunohistochemistry and MMTC showed that increasing stress periods significantly decreased GRP78 expression, although ATF4 and CHOP protein expression significantly increased. Stress resulted in pathological changes and significant dynamic changes because of endoplasmic reticulum stress in rat hypothalamic neurons. These results suggested that the endoplasmic reticulum stress PERK-ATF4-CHOP pathway may be associated with hypothalamic neuronal injury.

## Introduction

Stress is characterized by a comprehensive response of the neuroendocrine system and other systems that regulate and deal with threatening stimuli (McEwen, 2007). The body attempts to minimize the potential impact of a threat by adjusting the metabolism of each system and increasing the ability of steady-state properties (McEwen and Wingfield, 2010). Moderate stress increases the body's ability to resist external risk factors, while excessive stress damages the body and results in various abnormal psychological and physiological changes (Tsigos and Chrousos, 2002; Marin et al., 2011).

Previous studies have shown that the hypothalamus-pituitary-adrenal (HPA) axis plays a critical role in the body's stress response. The hypothalamus is activated by various signaling pathways induced by different external stimuli, which then further acts on the pituitary by releasing corticotropin-releasing hormone (CRH). An activated pituitary releases adrenocorticotropin (ACTH), which activates the adrenal cortex and facilitates the synthesis and release of glucocorticoids. Glucocorticoids enable the body to resist external risk factors by adjusting energy and metabolism.

The hypothalamus, which is the center of the HPA axis, plays an extremely important role in the regulation of glucocorticoids in resisting external stimuli. Previous studies have shown that long-term stress exposure leads to declines in organism resistance and different degrees of pathological injuries (Vyas et al., 2002; McLaughlin et al., 2007). However, there is no detailed report on whether different durations of stress exposure result in pathological changes in hypothalamic neurons.

The endoplasmic reticulum (ER) is the primary site for protein synthesis, glycosylation, folding, and secretion, as well as nascent protein transport (Marciniak and Ron, 2006; Ron and Walter, 2007; Nakayama et al., 2008). Dysfunction of ER or a mass of generated materials such as reactive oxygen and calcium ions under stressful conditions, can lead to accumulation of unfolded or misfolded proteins in the ER lumen, which induces endoplasmic reticulum stress (ERS) (Boyce and Yuan, 2006). Perturbations causing ERS result in the accumulation of unfolded and misfolded proteins in the ER and the initiation of a series of complex signal transduction cascades, known as the unfolded protein response (UPR). UPR is a relatively conservative and protective ERS response that has evolved over time. Protein kinase RNA (PKR)-like endoplasmic reticulum kinase (PERK) is an important sensing element for ERS (Hetz, 2012). When ERS occurs, the activated PERK induces the downstream signaling pathway to inhibit protein translation, thus restoring ER homeostasis (Wang et al., 2016). Conversely, if ERS is too long and/or severe, ATF4 is activated by the PERK signaling pathway (Harding et al., 2000), which in turn drives CCAAT/-enhancer-binding protein homologous protein (CHOP) transcription. Continued expression of CHOP can then induce cell death (Pillai, 2005; Namba et al., 2009).

As broadly documented in the literature (Doyle et al., 2011; Yuan et al., 2011; Hoozemans et al., 2012), ERS takes part in the response to cell and tissue injury, but is also linked to neuronal death in various neurodegenerative diseases. However, it remains unclear whether injured hypothalamic neurons result in disordered HPA axis function or whether the PERK-ATF4-CHOP signaling pathway is associated with the injury process after stress exposure. For the present study, we successfully established rat models with different durations of stress exposure to investigate serum glucocorticoid levels, pathological changes of hypothalamic neurons, and ERS-related protein changes to provide morphological evidence of the role of the PERK-ATF4-CHOP pathway in pathological changes of hypothalamic neurons.

## Materials and methods

### Animals

Adult, male, Sprague-Dawley (SD) rats (Experimental Animal Center, Hebei Medical University, China), weighing 250 ± 20 g, were allowed to adapt to a 12/12-h light/dark cycle, with *ad libitum* access to food and water. This study was approved by the Institutional Review Board for Animal Experiments at Hebei Medical University. Every attempt was made to reduce the number of animals and to minimize pain and suffering. The rats were randomly divided into the following groups: Control; 1, 3, 7, 14, and 21 days of restraint stress (RS) combined with ice water swimming (IS) groups (RS+IS group) (*n* = 6 rats per group).

### Restraint stress and ice water swimming protocols

The restraint stress protocol was adapted from a previously described method (Imbe et al., 2012); the rats could stretch their legs, but could not move within the restrainers. The rats were placed in the restrainer with no access to food and water for 8 h (from 8:00 to 16:00) each day. Then the restricted stress rats were placed in ice water to swim for 5 min each day. The stress-inducing exercises lasted for 1, 3, 7, 14, and 21 days. The control group rats were left in the cages for the same time without food and water. During the rest period, all rats were provided food and water *ad libitum*.

### Enzyme-linked immunosorbent assay (ELISA)

Serum glucocorticoid (Uscn Life Science Inc., Wuhan, China) levels were measured using an ELISA Complete Kit as per the manufacturer's instructions. Rat sera were collected from abdominal aorta blood.

Briefly, a standard solution or rat sera (glucocorticoid; 1:100) were added to each well and incubated at 37°C for 60 min. After washing three times with PBST (0.05%, Tween 20), horseradish peroxidase (HRP)-labeled anti-rat glucocorticoid and melatonin secondary antibodies were added and incubated at 37°C for 1 h. After removing the liquid from all wells, the wells were washed three times and color reactions were developed with 3, 3′, 5, 5′ tetra-methylbenzidine (TMB) color development solution. After the wells reacted for 15 min at room temperature in the dark, sulfuric acid (2 mmol/10 ml) was added. The plates were read at 450 nm using an ELISA reader (Multiskan MS photometer type 352, Labsystems, Helsinki, Finland). The final hormone concentration was calculated based on a standard curve constructed using the recombinant hormone standard.

### Tissue preparation

Tissue used for staining was harvested and fixed immediately in 10% formalin. Brain slices beginning at –1.80 mm from bregma were obtained using a stereotaxic atlas (Paxinos and Watson, 2007). The tissue was subsequently dehydrated in a graded ethanol series and embedded in paraffin. For hypothalamus analysis, consecutive 6-μm-thick coronal section with the largest hypothalamus area were collected that corresponded to −3.0 mm from bregma, as per the Paxinos and Watson atlas. Sections were prepared for thionine staining and immunohistochemical staining, and examined under a light microscope (Olympus IX71; Olympus, Tokyo, Japan).

### Immunohistochemistry

Deparaffinized sections were pretreated using microwave antigen retrieval, followed by incubation in 3% H_2_O_2_ in cold methanol for 30 min, and goat serum for 30 min. Next, the tissues were incubated overnight at 4°C with monoclonal antibodies specific for rabbit NeuN, GRP78, ATF4, and CHOP (1:100). The tissues were then incubated for 1 h with biotinylated secondary antibody and subsequently with horseradish peroxidase (HRP)-conjugated biotin for 30 min. Finally, 3, 3′-diaminobenzidine (DAB) was used as the chromagen. The tissues were counterstained with hematoxylin to visualize locations in the sections.

### Cell counting

Six rats from each group were used for morphological observation and data analysis. According to the stereotaxic atlas (Paxinos and Watson, 2007), the largest hypothalamus area was accurately exposed. Using the serial section technique, we took one out of every five sections and selected a total of three sections for each rat. With microscopy based multicolor tissue cytometry (MMTC), we evaluated the percentage of positive cells in the whole hypothalamic area of each section. The data of each rat was derived from the average of those three sections.

MMTC has been used by previous researchers (Ecker et al., 2006; Hamzei et al., 2016) and has the advantage of being more objective than the subjective assessment by an investigator. Sections were analyzed at × 100 field view using a Tissuefax Plus system based on Zeiss® AxioImagerZ2 Microscope (Jena, Germany). Images were acquired with the TissueFaxs (Tissue-Gnostics®, Vienna, Austria) software. The percentage of NeuN-, GRP78-, ATF4-, and CHOP- positive immunostaining cells in the largest hypothalamus area was quantified using HistoQuest® (Tissue-Gnostics) software.

HistoQuest® is an analytical tool used to quantify immunostaining based on single cells using the cell specific nucleus structure as the primary identification marker (hematoxylin), followed by an automatic segmentation of the immunostaining confined to the corresponding nucleus. A ring mask around this nucleus is interactively defined and set as parameter for sections stained with a certain marker-specific channel named single reference shade. The brown staining caused by chromogen (3,3′- diaminobenzidine, DAB) is automatically separated from the blue hematoxylin staining into their optical density counterparts. The mean optical density per cell is quantified by the segmentation method.

Region of interest was defined for the largest hypothalamus area. Identification of neurons was accomplished through morphometric parameters such as the nuclear size, shape. A background threshold for hematoxylin staining was determined interactively. Immunostaining cutoffs were determined as well (this tool differentiates between positive and negative cells; these were set in the dot blots). All images were acquired with the same setting parameters. The representative brown color (DAB chromogen) was picked by the color picker tool. Positive staining cells in the largest hypothalamus area were shown in the scatter gram of forward gating tool. The raw data of the analysis were imported into SPSS 21.0 (IBM, Armonk, NY, USA) for further statistical analysis. The number of NeuN-, GRP78-, ATF4-, and CHOP- positive immunostaining cells was divided by the total number of hypothalamic neurons, yielding a percentage of positive cells.

### Statistical methods

Using the method of Kolmogorov-Smirnov Test, the data was normal distribution among all groups (*P* > 0.1). The results are presented as mean ± SEM. Because of normal distribution in all samples, statistical analysis was performed using one-way ANOVA. *Post-hoc* LSD- *t* tests were used when comparisons were restricted to two experimental groups. The threshold for statistical significance was defined as *P* < 0.05.

## Results

### Measurement of serum glucocorticoid level by ELISA

**Figure 1:**
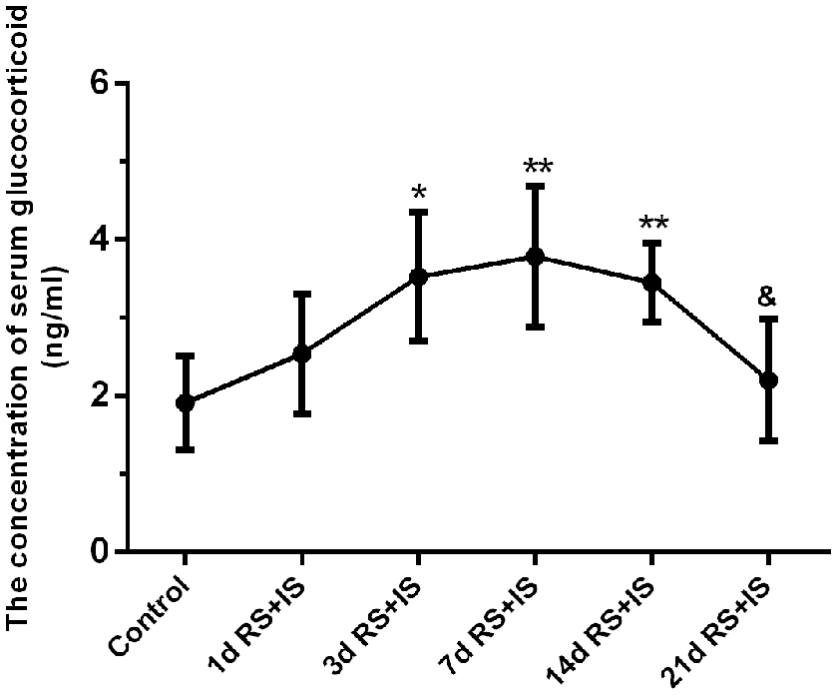
**Effect of different durations of stress on serum glucocorticoid levels**. Compared with the control group, the level of serum glucocorticoid significantly increased after stress exposure for 3 days, peaked at 7 days, and remained high at 14 days. However, with prolonged stress exposure, serum glucocorticoid level significantly decreased at 21 days compared with 14 days. The results are shown as mean ± SEM, ^*^P < 0.05, ^**^P < 0.01 vs. control group. ^&^*P* < 0.05 vs. RS+IS group at 14 days. d, day(s); RS+IS, restraint stress combined with ice water swimming.

ANOVA for serum glucocorticoid levels revealed that there were significant difference among the groups [*F*_(5, 30)_ = 6.073; *P* = 0.001]. Compared with the control group (2.06 ± 0.25), serum glucocorticoid levels were significantly upregulated in the RS+IS group at 3 days (3.51 ± 0.30, *P* < 0.05), peaked at 7 days (3.73 ± 0.34, *P* < 0.05), and maintained a high level at 14 days (3.42 ± 0.19, *P* < 0.05). However, with prolonged stress, levels significantly decreased by 21 days (2.28 ± 0.29, *P* < 0.05) compared with 14 days (Figure 1).

### Thionine staining shows pathological changes in hypothalamic neurons

**Figure 2:**
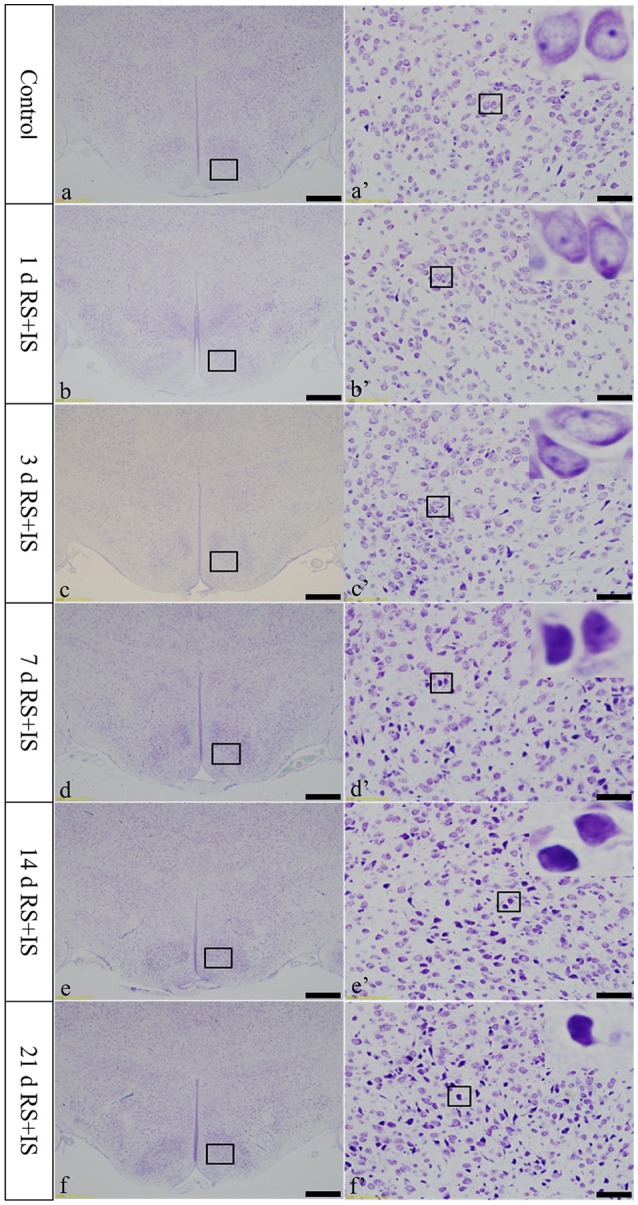
**Thionine staining of the hypothalamus**. (a'–f') Are magnified areas of (a–f), respectively. High-power photomicrographs in the right corners of (a'–f') are enlarged from rectangles in the low-power photomicrographs. With increasing periods of stress exposure, Nissl body structures are not clear, and neurons are pyknotic and deeply stained. Bars = 100 μm in (a–f); Bars = 50 μm in (a'–f'). d, day(s); RS+IS, restraint stress combined with ice water swimming.

In the control group, the neuronal structures were clear, and Nissl bodies were evenly distributed in the cytoplasm. In the RS+IS group, there were no significant changes in Nissl bodies at 1 and 3 days. However, edema was visible in the neurons and Nissl bodies were not clear at 7 days. After 14 days of stress expression, some Nissl bodies disappeared, and pyknotic neurons were visible. Cellular damage was more obvious at 21 days (Figure 2).

### Changes in NeuN-positive cells in the hypothalamus

**Figure 3:**
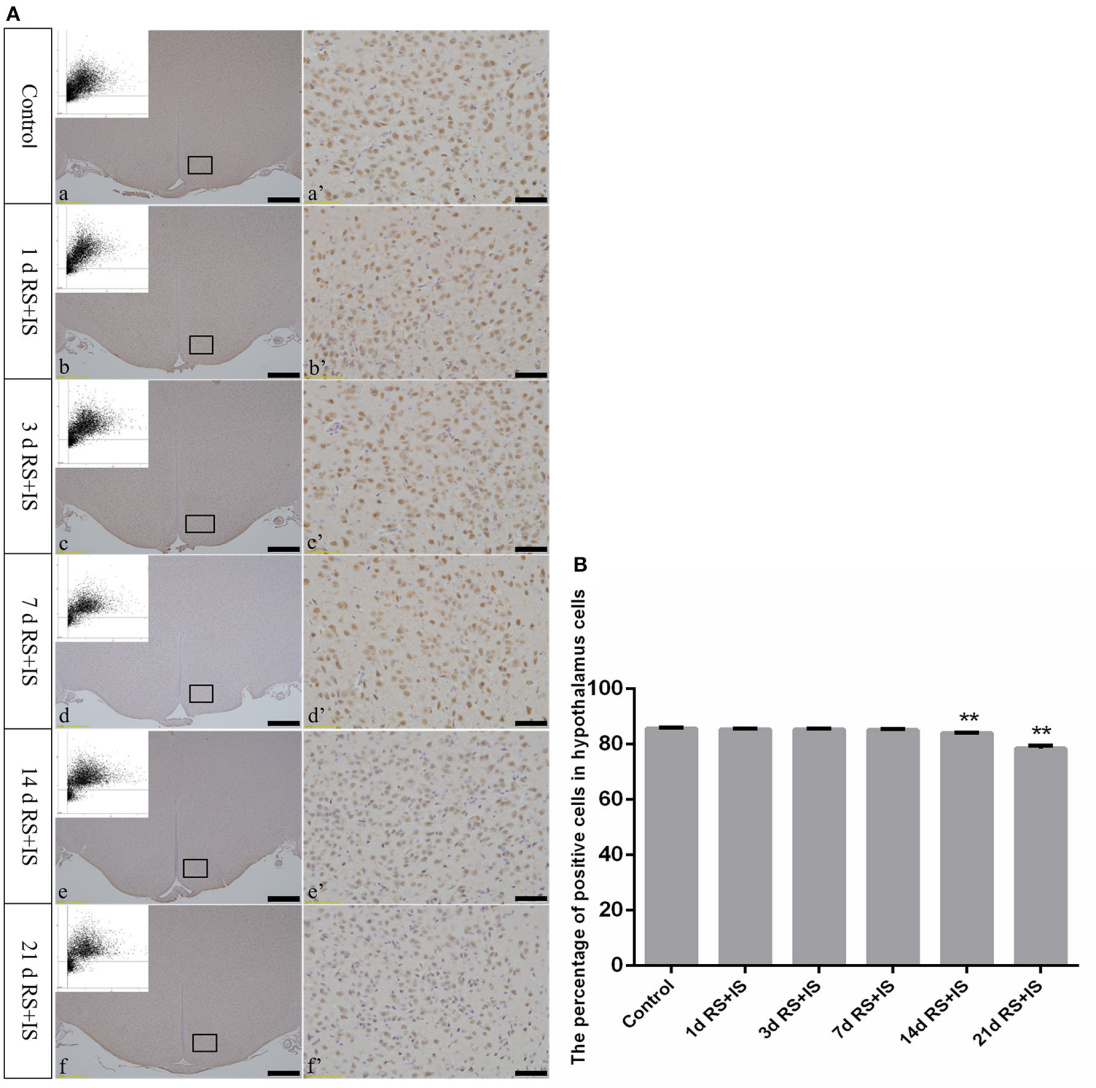
**(A)** Representative images showing NeuN immunohistochemistry in the hypothalamus. (a'–f') Are magnified areas of (a–f), respectively. Representative images obtained by microscopy-based multicolor tissue cytometry (MMTC) are shown in the left corners of (a–f). Bars = 100 μm in (a–f); Bars = 50 μm in (a'–f'). **(B)** Quantitative MMTC analysis. The data are shown as mean ± SEM, ^**^P < 0.01 vs. control group (*n* = 6). d, day(s); RS+IS, restraint stress combined with ice water swimming.

NeuN, which is expressed in the nucleus, was stained brown by immunohistochemical staining. With increasing stress duration, the number of NeuN-positive cells decreased (Figure 3A). HistoQuest software was used to quantify the number of NeuN-positive cells in the hypothalamus, showing there were significant difference among the groups [*F*_(5, 30)_ = 209.620; *P* < 0.001], but showed no change in the number of NeuN-positive cells in the RS+IS group at 1 day (85.34 ± 0.39, *P* > 0.05), 3 days (85.39 ± 0.20, *P* > 0.05), and 7 days (85.12 ± 0.44, *P* > 0.05) compared with the control group (85.74 ± 0.32). However, the number of NeuN-positive cells significantly decreased in the RS+IS group at 14 days (84.07 ± 0.09, *P* < 0.05) and 21 days (78.45 ± 1.08, *P* < 0.05) (Figure 3B).

### GRP78, ATF4, and CHOP protein expression in the hypothalamus

**Figure 4:**
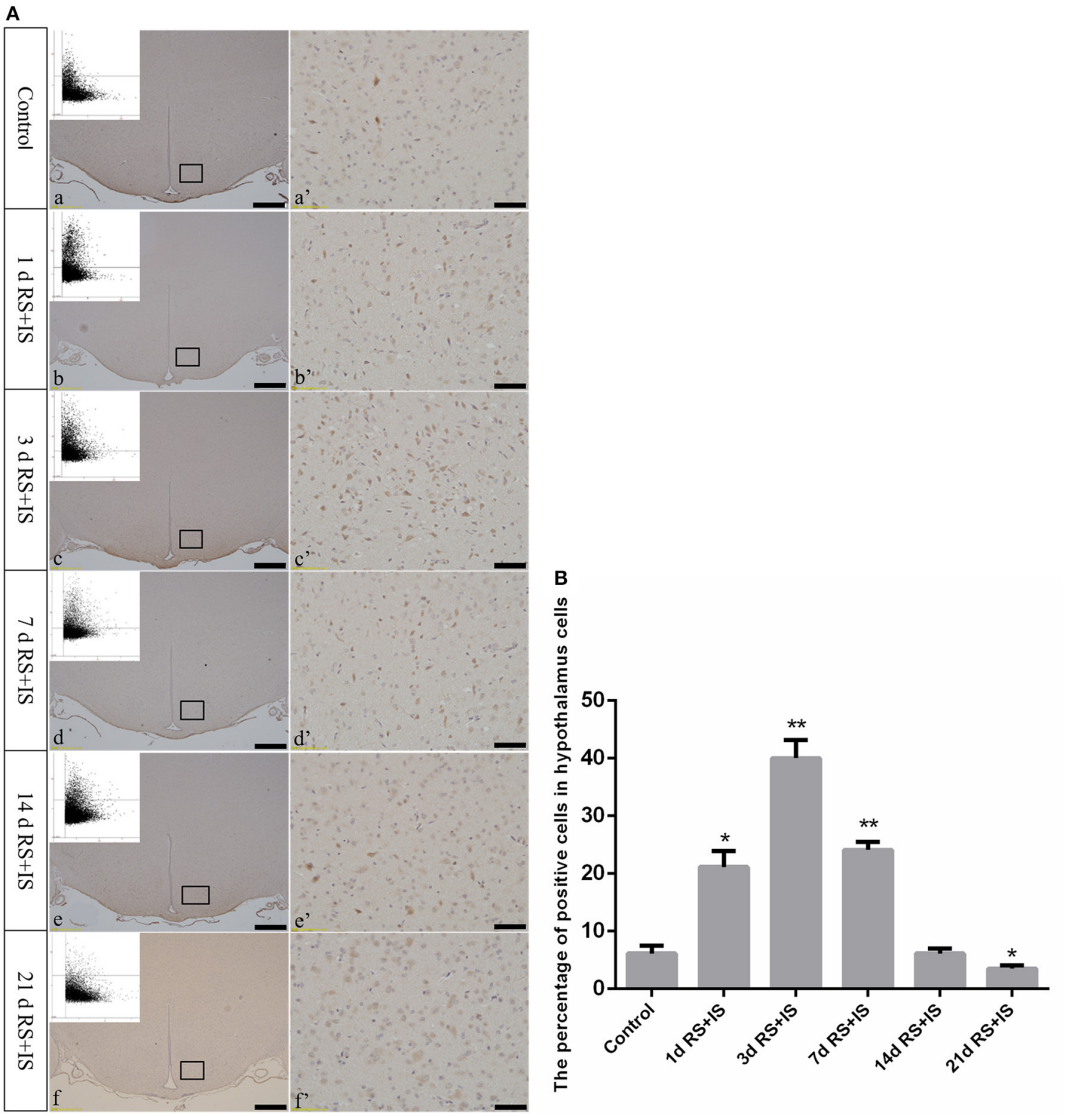
**(A)** GRP78 immunohistochemistry in the hypothalamus following different durations of stress exposure. (a'–f') Are magnified areas of (a–f), respectively. Representative images obtained by microscopy-based multicolor tissue cytometry (MMTC) are shown in the left corners of (a–f). Bars = 100 μm in (a–f); Bars = 50 μm in (a'–f'). **(B)** Quantitative MMTC analysis. The data are shown as mean ± SEM, ^*^P < 0.05, ^**^P < 0.01 vs. control group (*n* = 6). d, day(s); RS+IS, restraint stress combined with ice water swimming.

**Figure 5:**
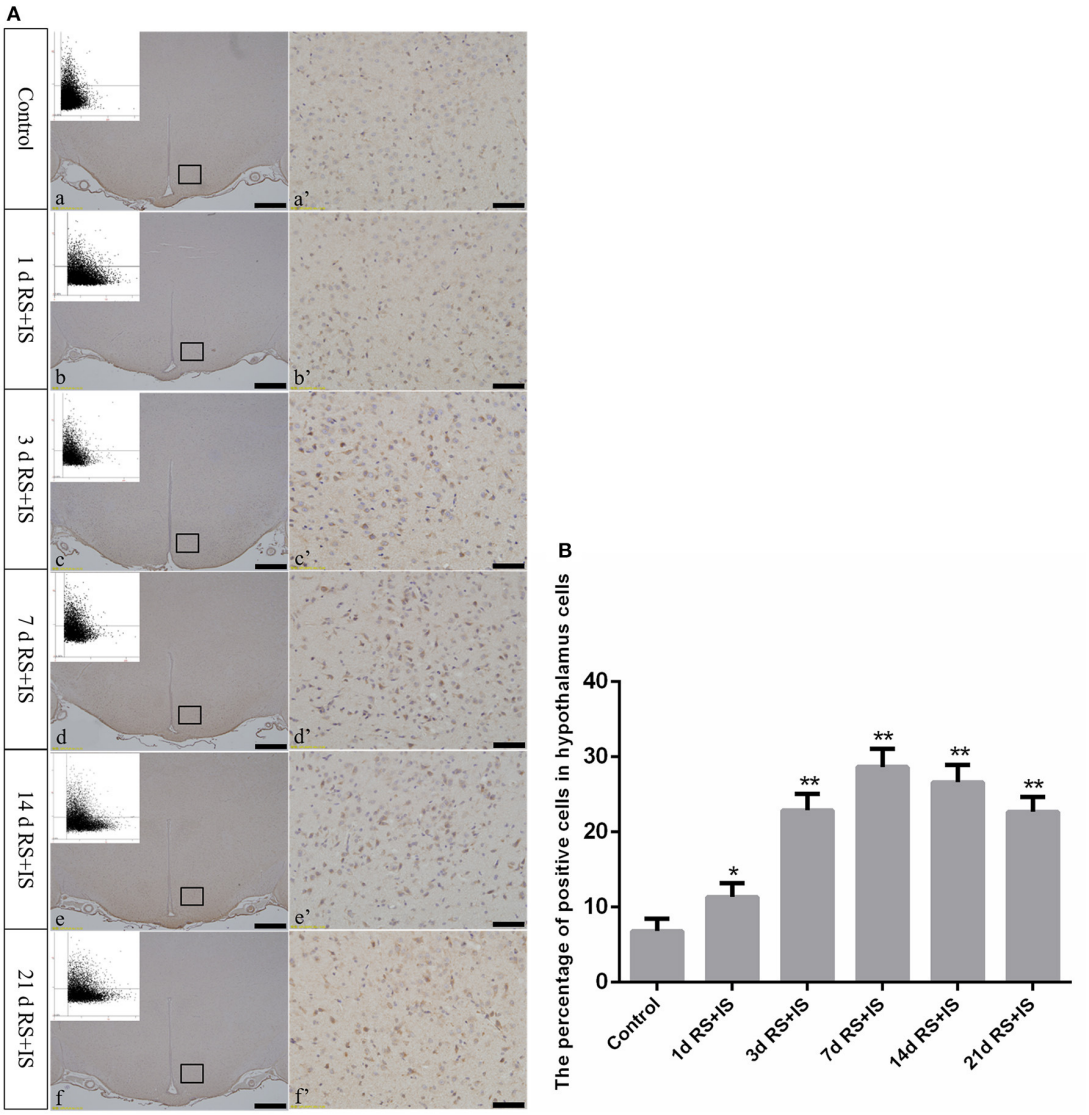
**(A)** Representative images showing ATF4-positive cell expression in the hypothalamus. (a'–f') Are magnified areas of (a–f), respectively. Representative images obtained by microscopy-based multicolor tissue cytometry (MMTC) are shown in the left corners of (a–f). Bars = 100 μm in (a–f); Bars = 50 μm in (a'–f'). **(B)** Quantitative MMTC analysis. The data are shown as mean ± SEM, ^*^P < 0.05, ^**^P < 0.01 vs. control group (*n* = 6). d, day(s); RS+IS, restraint stress combined with ice water swimming.

**Figure 6:**
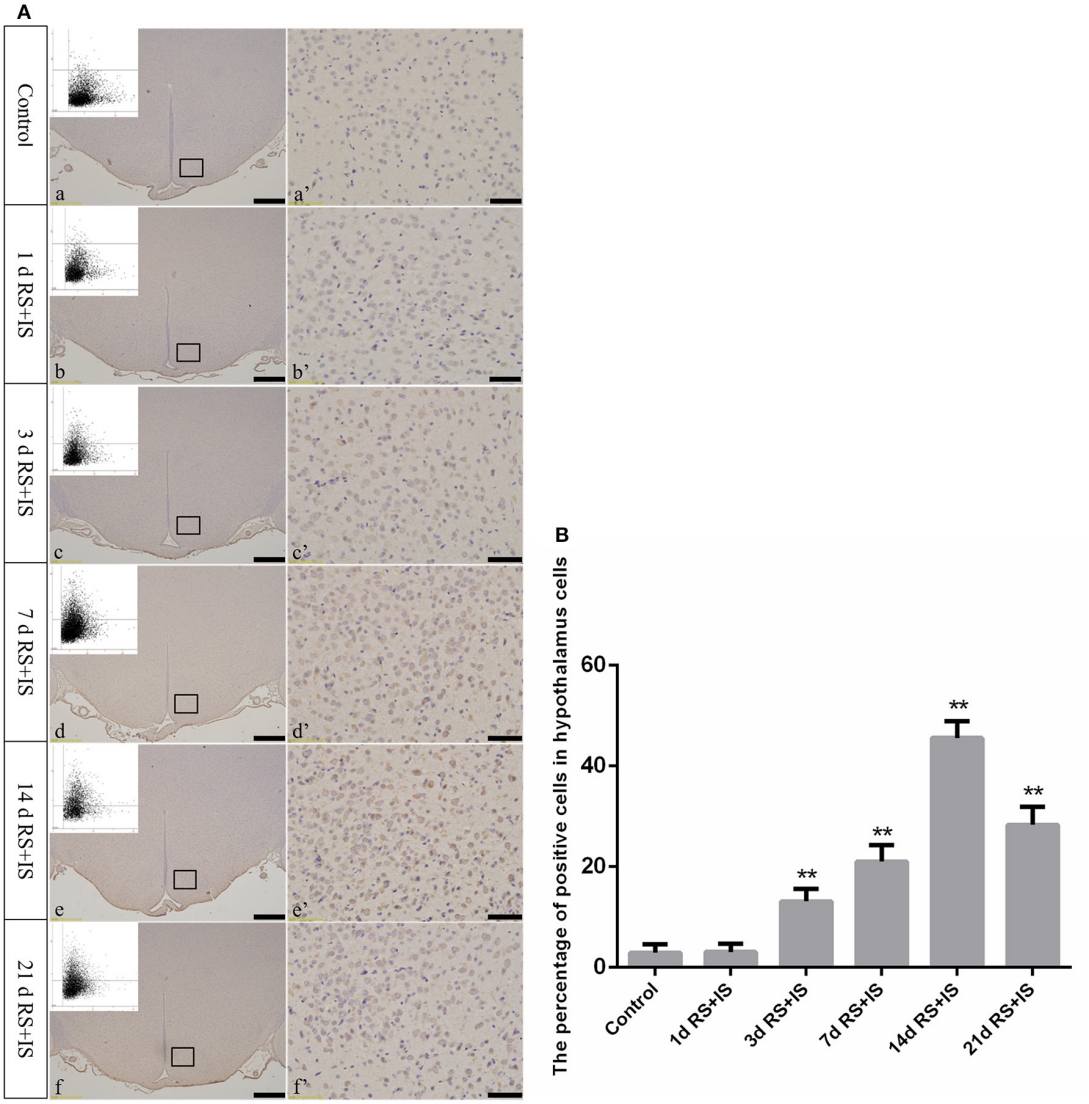
**(A)** Representative images showing CHOP immunohistochemistry in the hypothalamus. (a'–f') Are magnified areas of (a–f), respectively. Representative images obtained by microscopy-based multicolor tissue cytometry (MMTC) are shown in the left corners of (a–f). Bars = 100 μm in (a–f); Bars = 50 μm in (a'–f'). **(B)** Quantitative MMTC analysis. The data are shown as mean ± SEM, ^**^P < 0.01 vs. control group (*n* = 6). d, day(s); RS+IS, restraint stress combined with ice water swimming.

GRP78, ATF4, and CHOP proteins were located in the cytoplasm and were stained brown by immunohistochemical staining. ANOVA for GRP78 positive cells in the hypothalamus showed that there were significant difference among the groups [*F*_(5, 30)_ = 406.965; *P* < 0.001]. Compared with the control group (6.13 ± 1.35), GRP78 expression was significantly upregulated in the RS+IS group at 1 day (21.15 ± 2.80, *P* < 0.05), peaked at 3 days (40.02 ± 3.14, *P* < 0.01), and remained at a high level at 7 days (24.10 ± 1.97, *P* < 0.05), although there was no difference at 14 days (6.13 ± 0.85, *P* > 0.05). With further prolonged stress, GRP78 expression significantly decreased at 21 days (3.51 ± 0.56, *P* < 0.05) (Figure 4). ANOVA for ATF4 positive cells in the hypothalamus showed that there were significant difference among the groups [*F*_(5, 30)_ = 133.292; *P* < 0.001]. Compared with the controls (6.80 ± 1.64), stress exposure cause significant increases in ATF4 expression at 1 day (11.34 ± 1.85, *P* < 0.05), 3 days (22.89 ± 2.18, *P* < 0.05), 7 days (28.66 ± 2.42, *P* < 0.05), 14 days (26.60 ± 2.29, *P* < 0.05), and 21 days (22.69 ± 1.97, *P* < 0.05) (Figure 5). ANOVA for CHOP positive cells in the hypothalamus showed that there were significant difference among the groups [*F*_(5, 30)_ = 262.928; *P* < 0.001]. CHOP protein expression remained unchanged after 1 day of stress (3.07 ± 1.58, *P* > 0.05) compared with the control group (2.92 ± 1.65). However, CHOP expression significantly increased after 3 days (13.14 ± 2.48, *P* < 0.01) and 7 days (21.03 ± 3.31, *P* < 0.01) of stress exposure, peaked after 14 days (45.55 ± 3.31, *P* < 0.01), and remained at a high level after 21 days (28.33 ± 3.56, *P* < 0.01) (Figure 6).

## Discussion

Stress is an inevitable life experience that causes disturbances to body homeostasis and different organs and systems, including the central nervous system (Linthorst and Reul, 2008). Acute stress has a positive impact on the body. However, repeated and long term stress stimulation is harmful, often resulting in varying degrees of injuries to each organ and leading to negative effects on body function and metabolism (Hetz, 2012). Previous studies have shown that prolonged stress is a critical risk factor for the onset of several neuropsychiatric disorders, such as anxiety, major depression, and other neurodegenerative diseases (Goldstein, 2011). Animal models are one of the most efficient ways to identify and study the underlying mechanisms of the effects of stress on the body. In the present study, the rat were exposed to restraint and ice-water swimming for a maximum of 21 days to mimic human characteristics of chronic psychological frustration and physiological stress and to determine the effects of stress on body function.

When the body is exposed to a variety of stimuli, the HPA axis becomes stimulated and ultimately releases large amounts of glucocorticoid from the adrenal cortex in response to external stimuli. Glucocorticoid levels are a good indicator of the body's ability to resist external stress. The present study demonstrated that prolonged stress exposure resulted in significantly increased serum glucocorticoid levels, suggesting an increased defense capability to external stimuli by regulating the HPA axis and ultimately maintaining homeostasis. Numerous studies have indicated that glucocorticoids play a critical role in maintaining cardiovascular stability, stabilizing lysosomal membranes, anti-inflammation, anti-shock mechanisms, and reducing bacterial endotoxin damage (Kasahara et al., 2015; Kadmiel et al., 2016). However, by increasing the stress period to 21 days, serum glucocorticoid levels significantly decreased compared with stress exposure of 14 days. This suggested that repeated and long term stress exceeded the body's ability to regulate and resist injuries, which may cause pathological changes in tissue and cells. Accordingly, thionine staining revealed morphological changes in hypothalamic neurons. These results demonstrated that prolonged stress exposure resulted in reduced Nissl bodies in hypothalamic neurons and pyknotic neurons in the RS+IS group at 14 and 21 days.

The neuron-specific marker, NeuN, was used to label and quantify hypothalamic neurons. Results showed that long term stress exposure contributed to a decreased number of hypothalamic neurons, suggesting that with increasing periods of stress exposure, hypothalamic neurons exhibit pathological changes and injury, which likely contributes to disordered HPA axis functions and a weakened body resistance.

The relationship between ERS and damage has been a sought-after topic in recent studies. ERS not only participates in a variety of neurodegenerative diseases (Doyle et al., 2011; Yuan et al., 2011; Hoozemans et al., 2012), but also plays a critical role in cognitive dysfunction induced by stress (Zhang et al., 2014; Wu et al., 2016). Moderate ERS is involved in mechanisms of cell protection, but excessive ERS results in damage. A mass of materials such as reactive oxygen and calcium ions generated when stress exposure, which result in amassing of unfolded or misfolded proteins in the ER and initiate ERS (Hanada et al., 2007). Meanwhile, the accumulating unfolded and misfolded proteins cause dissociation of GRP78 from the ER transmembrane effector protein. GRP78, a specific marker of ERS activation, is only expressed in the ER and plays a cellular protective role. It not only determines UPR initiation, but also plays a key role in promoting maturation of unfolded proteins and maintaining ER homeostasis (Lee, 2001, 2005; Hendershot, 2004). In the present study, GRP78 expression exhibited a trend of first increasing and then decreasing, although by 21 days, expression was significantly decreased compared with the control group. These results suggested that the protective effect of GRP78 on hypothalamic neurons was significantly weakened with prolonged stress duration. Cells were not able to restore homeostasis, and a series of downstream signaling pathways were activated to initiate and induce cellular injury and death.

CHOP, also known as growth arrest and DNA-damage-inducible gene 153 (GADD153), is a critical mediator of ERS-induced cell death (Szegezdi et al., 2006). CHOP is expressed at low levels under physiological conditions, but is dramatically upregulated during severe and prolonged ERS and plays a crucial role in cell arrest and inducing cell death (Oyadomari and Mori, 2004; Tajiri et al., 2004). The PERK-ATF4 pathway plays a primary role in inducing CHOP transcription (Fels and Koumenis, 2006). When ERS expression is excessive, the upstream regulatory sequence of the 5′ terminal that contains an open reading frame gene becomes activated, which inhibits eIF2α-dependent protein translation (Schröder and Kaufman, 2005) and sensitizes the downstream transcription factor ATF4. Activated ATF4 induces and increases CHOP expression by increasing its transcription (Pillai, 2005; Xu et al., 2005). In this study, the PERK-ATF4-CHOP pathway was activated by stress, resulting in sustained increased levels of ATF4 and CHOP protein expression. CHOP overexpression could contribute to cell injury and death, which is consistent with our findings showing pathological changes in hypothalamic neurons after 14 days of stress exposure. These data suggested that the PERK-ATF4-CHOP pathway was associated with hypothalamic neuronal injury induced by long term stress stimulation.

In conclusion, this study clearly indicated that injured hypothalamic neurons resulted in disordered HPA axis function and the PERK-ATF4-CHOP signaling pathway is involved in the injury process after stress exposure. We believe these novel findings will provide morphological evidence of the mechanisms involved in stress-induced neuron injury.

## Author contributions

SY, WS, HW, CM, XZ, SW, YL, and BC wrote the paper and designed and performed the experiments. SY, WS, and HW carried out statistical analyses and organized the data. XZ and SW created the figures. CM, YL, and BC supervised the research design and revised the manuscript. All authors read and commented on the manuscript.

## Funding

This work was supported with funds from the Key Projects of National Natural Science Foundation of China (81430047) and the National Natural Science Foundation of China (81571850).

### Conflict of interest statement

The authors declare that the research was conducted in the absence of any commercial or financial relationships that could be construed as a potential conflict of interest.
